# High Bifurcation of the Brachial Artery: An Embryological Overview

**DOI:** 10.7759/cureus.7097

**Published:** 2020-02-25

**Authors:** Gregory Tsoucalas, Anna Eleftheriou, Eleni Panagouli

**Affiliations:** 1 Anatomy, School of Medicine-Democritus University of Thrace, Alexandroupolis, GRC; 2 Anatomy, Democritus University, Alexandroupolis, GRC; 3 Anatomy, National and Kapodistrian University of Athens, Athens, GRC

**Keywords:** axis artery, radial artery, ulnar artery, surgery, radiology

## Abstract

The brachial artery is the main artery of the arm and constitutes the continuation of the axillary artery. It gives off two terminal branches, the radial and ulnar arteries. According to the literature, the brachial artery might present a deviation from the normal pattern in 20% of the cases. High bifurcation of the artery seems to be the most common variation and may result in a series of complications during surgery and interventional radiology. An embryological overview is necessary for a better understanding of this variant. The brachial artery is being developed during embryonic life by the main trunk of the axis artery. The superficial brachial artery is also an important stable fetal vessel for normal arterial morphogenesis of the upper limb.

## Introduction and background

Upper limb arterial variations are often encountered, either in routine autopsies or during daily clinical practice. Individual descriptions of such variants have appeared since the 17th century, though without a systematic description until Quain’s work in 1844, which was later followed by other studies [1-2]. According to available medical literature, variations in the branching pattern of the main arteries of the upper limb are discovered in 25% of the studied cases. Variations of the radial and ulnar arteries are frequently observed as well as variations of the brachial artery, albeit less often [3].

The brachial artery (BA) is the continuation of the axillary artery in the arm. It begins at the distal border of the tendon of teres major muscle and terminates by dividing into the radial (RA) and ulnar (UA) arteries, about 1 cm distal to the elbow joint. The RA is smaller in length than the UA. However, it seems to be a more direct continuation of the BA. Variations of the BA are considered common and are presented in Table 1. It could be divided proximally into two trunks, which then reunite [4]. The BA could also follow a superficial course, coursing in front of rather than behind the median nerve. This superficial brachial artery has an incidence, which varies from 3.6% to 9.6% [5]. Another frequent variation of the BA seems to be the division of the artery into its terminal branches at a higher than the expected level. This high division, usually named "high bifurcation of the brachial artery" (HBBA), might occur in several forms. Most often, the RA arises first, presenting a high origin (brachioradial artery) while the UA and the common interosseous artery (CIA) continue as a common trunk [4-7]. The BA might present a deviation from the normal anatomy pattern in 20% of the cases. HBBA is reported to be one of the most common variations, with an overall incidence of about 8% [7-8]. Recent studies demonstrated that an HBBA presents various incidences among sexes and racial groups [9].

**Table 1:** Variations of the brachial artery Variations of the brachial artery and the corresponding incidences as they are represented in the literature [5-6,8]

Variations of the brachial artery	
Variation	Incidence
Superficial brachial artery	3.6% to 9.6%
High bifurcation of the brachial artery	8%
High origin of radial artery	15%
High origin of ulnar artery	2%
High division of a superficial brachial artery	<1%

As various interventional surgical and radiological procedures are performed in the upper extremity, knowledge of the arterial variations of this region is of great importance for clinicians in order to avoid injuries and fatal results for their patients. Inappropriate cannulation in cases of aberrant locations of the arteries of the upper arm may result in thrombosis, gangrene, or, in extreme cases, even limb loss. Cannulation of the BA in clinical practice is performed for the diagnostic and therapeutic management of many pathological entities, like coronary artery disease, aortic and peripheral vascular disease, and chronic renal failure. HBBA might cause major clinical implications, including the high failure rate and decreased functional patency of an arteriovenous fistula [7-8]. An interesting study was carried out between January 2007 and June 2008 in London's King’s College Hospital by Domenico Valenti and colleagues, who studied the correlation between HBBA and the failure of the outer arteriovenous communication (A-V shunt). This study showed an increased probability of failure with an incidence of 45% in patients presenting an HBBA in comparison with a patient presenting a normal branching pattern who suffered an A-V shunt only in 23% of the cases [10]. Branching variations of the upper arm in simultaneously existing variations in the brachial plexus may result in an incomplete block of the plexus [11]. Furthermore, variations in the BA pattern may cause implications in planning and conducting flap harvesting during reconstructive surgeries and in arteriography [12].

Thus, surgeons and interventional radiologists must be familiar with the existence of the plethora of arterial anomalies, which may complicate their procedures. Clinical anatomy and branching anatomy variations of the human vessels are paramount [13]. The purpose of the present study is to provide an embryological overview in order to strengthen knowledge related to an HBBA and to highlight its clinical implications.

## Review

Arterial development of the upper limb

**Figure 1:**
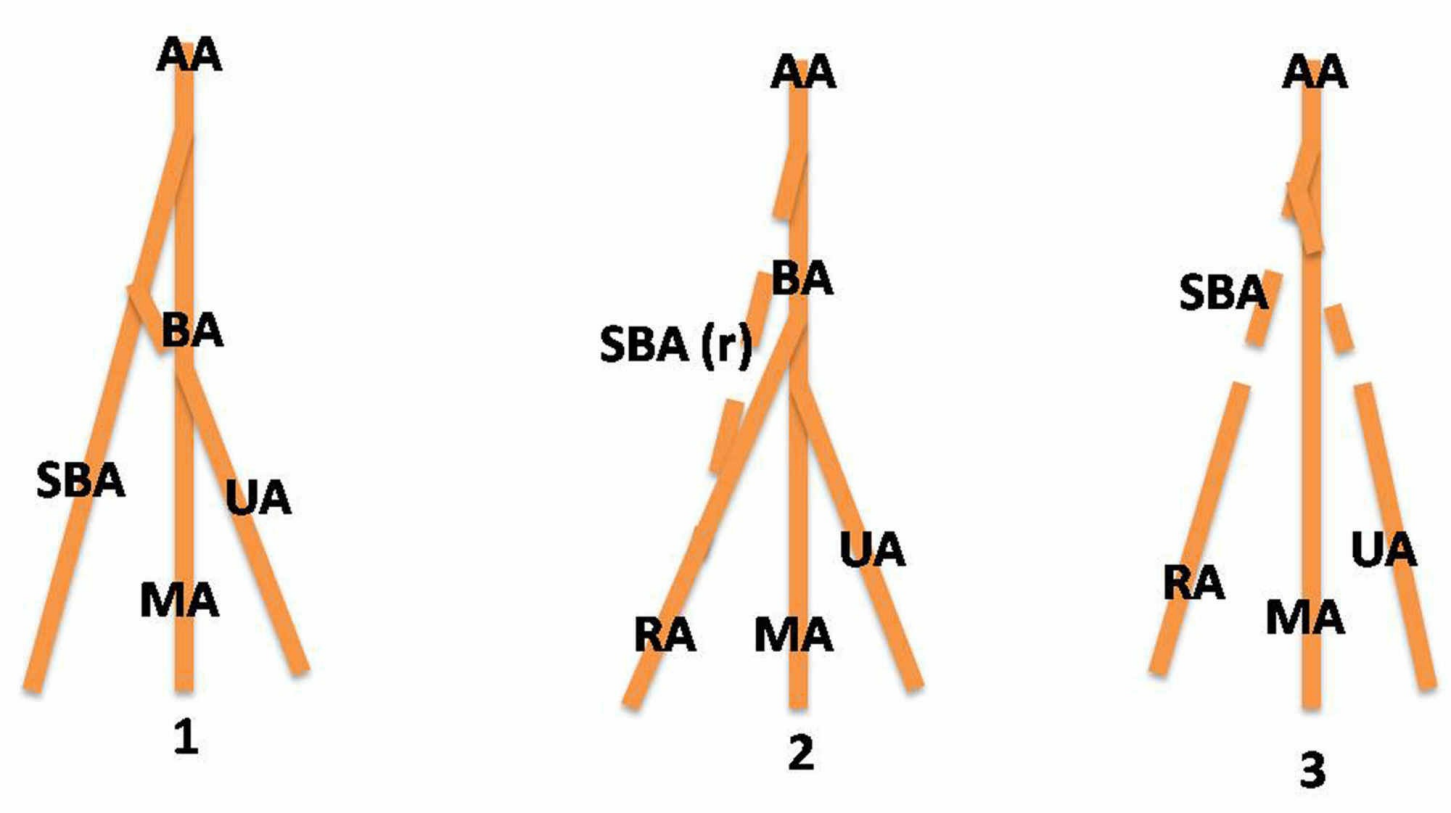
Embryologic development of upper limb arteries and possible explanation of high bifurcation of the brachial artery The main trunk of the axis artery (AA) forms the brachial artery. The superficial brachial artery (SBA) gives branching to the ulnar artery (UA) and continues in the forearm as part of the radial artery (RA) while the rest of it regresses [1-2]. In cases of non-regression of the SBA, the artery continues as the RA and gives rise to the UA, resulting in the high bifurcation of the brachial artery. SBA (r): superficial brachial artery regressed, MA: median artery

The upper extremity arterial system demonstrates an oversized number of variations, which probably are due to their complex embryonic development. The upper extremity arterial system demonstrates a large number of variations, which probably are due to their complex embryonic development. In order for a clinician to understand the mechanisms of the observed variants of HBBA (Figure 1), a brief reference should be made to the embryonic morphogenesis of the upper limb vessels. Each upper limb of the embryo is supplied by an axis artery (AA) that is derived from the seventh intersegmental (subclavian) artery. The AA develops in growth distally along the ventral axial line and terminates in a palmar capillary plexus in the hand. The main trunk of the AA forms the axillary artery (AXA), the BA, the anterior interosseous artery (AIA), and the deep palmar arch [14]. The superficial brachial artery (SBA) is a stable fetal vessel that plays an important role in the normal arterial morphogenesis of the upper limb. It has two final branches, a medium, which is the superficial artery of the forearm, and a collateral one, which continues in the forearm as part of the RA (Figure 1). The superficial artery of the forearm is divided into two final branches, median and ulnar [15]. Each of these branches is anastomosed with a corresponding branch of the primitive axial, whose origin is in the medial artery (MA) and UA, respectively. Gradually, the branches with deep origin dominate hemodynamically, and the superficial artery of the forearm, along with the pro-anastomotic part of its final branches, subverts (Figure 1). Therefore, two distinct parts may be distinguished in both the MA and the UA, a proximal or a depth corresponding to branches with an origin from the primary axial artery and an upper or superficial one that represents the post-anastomotic parts of the final branches of the superficial artery of the forearm. The RA usually develops similarly to the MA and BA. That is, the final collateral branch of the SBA is anastomosed by a branch for the deep origin of the RA from the primitive AA. The hemodynamic dominance determines the involution of the superficial parts of the arteries that are located near the anastomosis while the upper parts remain as part of the RA [15-16]. An unusual induction and branching of primitive vascular plexuses, lead by vascular growth factors (such as vascular endothelial growth factor) and developmental hemodynamics, may result in variations such as a high bifurcation of the brachial artery [17].

This description is in accordance with those given by Senior in 1926 and Singer in 1933 [18-19]. Similar patterns have also been described by Lippert and Pabst and later studies [16,20-21]. Sieger et al., in 2019, emphasized the importance of angioblasts in the vascular pathway and highlighted their role along with variable developmental hemodynamics in the emergence of unusual arterial patterns [17]. Abnormalities in this process of morphogenesis during the embryological life of the human embryo may result in a series of variations both concerning the origin and the course of the vessels of the upper limb [18-19].

Surgeons and interventional physicians should have a thorough knowledge of brachial artery variations. HBBA might cause confusion during angiographic procedures. The unusual position of the artery makes its recognition and catheterization difficult. The superficial course of the brachial artery might lead to serious injury and accidental intra-arterial injection. On the other hand, this abnormal course makes arterial grafting and cardiac catheterization easier [22]. The abnormal arterial pattern of the upper arm, such as HBBA, might also complicate surgical procedures such as distal biceps tendon repair [23].

## Conclusions

Exact knowledge of the anatomy of the arteries of the upper limb and their variations is necessary for designing appropriate invasive or surgical treatments for arm, forearm, and hand disorders. Comprehension of the embryological development of the HBBA branching pattern would help surgeons and radiologists towards that end.

## References

[1] Keen JA (1961). A study of the arterial variations in the limbs with special reference to symmetry of vascular patterns. Am J Anat.

[2] Hazlett JW (1949). The superficial ulnar artery. Can Med Assoc J.

[3] Adachi A, Hasebe K, Daigaku KT, Kenkyūsha Kenkyūsha (1928). Das Arteriensystem der Japaner Vol 1 [Book in German]. Kyoto, Japan.

[4] Standring S (2008). Gray’s Anatomy. The Anatomical Basis of Clinical Practice. http://www.ajnr.org/content/26/10/2703.short.

[5] Rodríguez-Niedenführ M, Vázquez T, Nearn L, Ferreira B, Parkin I, Sañudo JR (2001). Variations of the arterial pattern in the upper limb revisited: a morphological and statistical study, with a review of the literature. J Anat.

[6] Tountas CP, Bergman BA (1993). Anatomic Variations of the Upper Extremity. Churchill Livingstone, Edinburgh, United Kingdom.

[7] Panagouli E, Anagnostopoulou S, Venieratos D (2014). Bilateral asymmetry of the highly bifurcated brachial artery variation. Rom J Morphol Embryol.

[8] Cherukupalli C, Dwivedi A, Dayal R (2007). High bifurcation of brachial artery with acute arterial insufficiency: a case report. Vasc Endovascular Surg.

[9] Pham XD, Kim JJ, Parrish AB, Tom C, Ihenachor EJ, Mina D, de Virgilio C (2016). Racial and gender differences in arterial anatomy of the arm. Am Surg.

[10] Valenti D, Mistry H, Junghans C, Haughey N, Freedman B, Tyrrell M, Valenti D (2009). High brachial artery bifurcation is associated with failure of upper limb autologous arteriovenous fistulae. J Vasc Surg.

[11] Claassen H, Schmitt O, Wree A, Schulze M (2016). Variations in brachial plexus with respect to concomitant accompanying aberrant arm arteries. Ann Anat.

[12] Hansdak R, Arora J, Sharma M, Mehta V, Suri RK, Das S (2015). Unusual branching pattern of brachial artery - embryological basis and clinicoanatomical insight. Clinica Terapeutica.

[13] Tsoucalas G (2018). Anatomy: an essential course for future surgeons. J Univers Surg.

[14] Singh V (2013). Textbook of Clinical Embryology. https://books.google.co.in/books?hl=en&lr=&id=ABBtAwAAQBAJ&oi=fnd&pg=PP1&dq=Textbook+of+Clinical+Embryology%2BSingh&ots=kU06gvCGvD&sig=RA-ZSHpylz3Z1VYqchKN8dF9xtc&redir_esc=y#v=onepage&q=Textbook%20of%20Clinical%20Embryology%2BSingh&f=false.

[15] Lanz J, Wachsmuth W Praktische Anatomie, Vol 3.

[16] Lippert H, Pabst R (1985). Arterial Variations in Man - Classification and Frequency.

[17] Sieger J, Patel L, Sheikh K, Parker E, Sheng M, Sakthi-Velavan S (2019). Superficial brachioulnar artery and its clinical significance. Anat Cell Biol.

[18] Senior HD (1926). A note on the development of the radial artery. Anat Rec.

[19] Singer E (1933). Embryological pattern persisting in the arteries of the arm. Anat Rec.

[20] Rodríguez-Niedenführ M, Burton GJ, Deu J, Sañudo JR (2001). Development of the arterial pattern in the upper limb of staged human embryos: normal development and anatomic variations. J Anat.

[21] Chakravarthi KK, Ks S, Venumadhav N, Sharma A, Kumar N (2014). Anatomical variations of brachial artery: its morphology, embryogenesis and clinical implications. J Clin Diagn Res.

[22] Natsis K, Piagkou M, Panagiotopoulos NA, Apostolidis S (2014). An unusual high bifurcation and variable branching of the axillary artery in a Greek male cadaver. SpringerPlus.

[23] Zeltser DW, Strauch RJ (2016). Vascular anatomy relevant to distal biceps tendon repair. J Shoulder Elbow Sur.

